# Exploring the effect of problem based facilitatory teaching approach on motivation to learn: a quasi-experimental study of nursing students in Tanzania

**DOI:** 10.1186/s12912-020-00509-8

**Published:** 2021-01-04

**Authors:** Walter C. Millanzi, S. M. Kibusi

**Affiliations:** Department of Nursing Management and Education, School of Nursing and Public Health, The University of Dodoma (UDOM), Dodoma, Tanzania

**Keywords:** Motivation, Problem-based, Learning, Facilitator, Teaching, Nursing, Pedagogy, Education, Medical, And curriculum

## Abstract

**Background:**

Academic motivation is most important as a key determinant of competent and motivated nurses who are often considered as frontline healthcare providers who devote most of their time taking care of clients and patients. However, most of them demonstrate remarkable differences in their academic performances during their schooling that might be due to the differences in their academic motivation and achievement motivation. There appears a growing concern to rethink the approaches on how nurses are prepared, explore, and test novel approaches for delivering the nursing curricula. This study tested the effect of Problem Based Facilitatory Teaching pedagogy on academic motivation among nursing students in Tanzania, higher learning institutions.

**Methods:**

A pre-post-test controlled quasi-experimental study of 401 purposively selected participants was conducted between February and June 2018. The study was not a clinical randomized controlled trial and thus it has not been identified in the title and no summary of trial design, its methods, results, and conclusion. The Auditing Inventory developed by the researcher measured the intervention and a Questionnaire titled “Academic Motivation Scale,” was adopted to measure academic motivation. Statistical Product for Service Solutions software program version 23 was used to perform descriptive analysis to establish participants’ sociodemographic profiles. Regression analysis was performed to determine the association between variables.

**Results:**

Findings revealed that 65.8% of participants were males. Post-test findings showed 70.3% of participants demonstrated the motive to learn contrary to 34.9% at baseline. The odds of an intervention to influence academic motivation among participants was higher than the control (AOR = 1.720; *p* < 0.05; 95%CI: 1.122, 2.635). However, the intervention demonstrated little influence on the extrinsic motivation to learn (AOR = 0.676, *p* > 0.05, 95%CI: 0.405, 1.129) and Amotivation to learn (AOR = 0.538, *p* > 0.05; 95%CI: 0.283, 1.022) compared to the control.

**Conclusion:**

The Problem Based Facilitatory Teaching pedagogy was a predictive factor to intrinsic academic motivation among nursing students. The approach demonstrated educational potentials to change the spectrum of nursing competency and quality of care to patients or clients. This study suggests problem-based facilitatory teaching pedagogy be integrated into the nursing curriculum in Tanzania as it is feasible.

## Contribution to the Literature

- Findings of this study enlighten the professional associations as welfare organs about the existing pedagogical gaps demonstrated by the lecture-based learning approach on motivating nursing students to learn so that its alternative problem based facilitatory pedagogy be incorporated and implemented in the nursing curriculum among Tanzanian higher training institutions
- Moreover, the findings of the study give light to program and curriculum developers about the importance of developing programs and curriculum in the nature of problem-based facilitatory pedagogy that emphasizes collaborative learning to promote nursing students’ motivation to learn. By so doing, it might ensure the production of the motivated nursing graduate who will be able to work independently in an ethical manner when providing health care services among people.
- Findings establish a vital knowledge that informs instructors on how to design and implement nursing curriculum courses in the nature of problem-based facilitatory pedagogy to promote nursing students’ motivation to learn and be interested in nursing programs.
- Researchers will also use the findings of this study as baseline data for further interventional studies and or projects.

## Background

Nurses are often considered as frontline healthcare providers who devote most of their time taking care of clients and patients [1]. Academic motivation is most important as a key determinant of competent and motivated nurses who are often considered as frontline healthcare providers who devote most of their time taking care of clients and patients [2]. However, most of them demonstrate remarkable differences in their academic performances during their schooling that might be due to the differences in their academic motivation and achievement motivation [3]. Nursing students’ differences in their academic and achievement motivation have been associated with the unethical and illegal practices, under standard care and malpractice they demonstrate in clinical settings when providing care to patients or clients [4].

Educators and other stakeholders in nursing education link unethical, illegal, and malpractices among nurses with the inadequate and improper nurse preparations in low and higher nursing training institutions, which are the potential academic environment to improve their academic motivation and achievement motivation [5]. Millanzi et al.*,* [4] uncovered that recent changes in nursing education and the community and stakeholders’ concerns about the quality of nursing care expected from competent nurses have led to the desire to rethink and provide meaningful teaching and learning, which motivates students to learn with minimal support from instructors. Motivation to learn is a measure of nursing competence, which indicates that professional nurses are prepared to resolve nursing problems in a rapidly changing environment [6].

Carroll [7] described the academic motivation and academic achievements to be two significant factors in the analysis of academic performance for students. Academic motivation refers to a student’s inner desire that guides behavior towards learning and academic achievements influenced by both, internal and external factors. Moreover, Khamoushi et al.*,* [8] defined academic motivation as a learner’s internal aspiration towards learning and academic achievements that can be influenced by both internal factors such as interest, reasons, or goals and external factors such as rewards, punishment, or material gain. This study believed that motivation plays a major role in explaining behaviors, predicting the effects of actions, and guiding behavior to achieve objectives.

Academic motivation does not only promote learning but also an intermediate impact on learning which helps students to have smooth relationships, decrease stress, increase creativity, and promote open learning [9]. Various scholars define academic motivation as the required stimulation to do the assignments, to achieve the goals, or to acquire a certain degree of competence in one’s work and consequently gain academic achievements [5, 10, 11]. It is a key factor in a student’s academic performance in a problem-based environment. Academic motivation can be classified as intrinsic motivation (to know), intrinsic motivation (toward accomplishment), intrinsic motivation (to experience motivation), extrinsic motivation (Identified), extrinsic motivation (Introjected), extrinsic motivation (External regulation), and Amotivation to learn.

Motivating nursing students to learn is an essential component to ensure competent graduates who can exhibit safe, ethical, and legal practice as a backbone and critical issue in the nursing education field [5, 10, 12]. The importance of motivating nursing students to learn has also been pinpointed by scholars such as Duiker [6] who found out that nursing student can demonstrate ethical and legal professional conducts when are well-formed in a well and organized motivating learning environment. The reviewed pieces of literature unfold that students’ motivation in education is often driven by two questions “Can I do this task?” (Beliefs on one’s capabilities, factors that cause success, and one’s low influence on success). Moreover, questions such as “Why am I doing this task?” (Task values-interestingness, importance, utility, goal orientation) can drive students’ motivation to learn.

The curricula in nursing institutions are challenged to motivate nursing students to acquire appropriate skills that will allow them to offer high-quality care to patients/clients through safe, ethical, and legal practices [13]. Challenges are still visible particularly on enhancing student’s intrinsic motivation (to know), intrinsic motivation (toward accomplishment), and intrinsic motivation (to experience motivation). Some literature acknowledges that the existing pedagogical approaches including the lecture method to have demonstrated remarkable contributions to improving extrinsic motivation (Identified), extrinsic motivation (Introjected), extrinsic motivation (External regulation), and Amotivation to learn among nursing students.

Some global initiatives including the introduction of competency-based curricula in nursing are being practiced to address the existing content and pedagogical gaps. Tanzania is among Sub-Saharan African countries that adopted competency-based curricula. However, no evidence shows how Health Science Colleges/Universities have changed to cater to new demands [1, 14]. Tutors and lecturers, still focus on developing course contents along with traditional instructional-based pedagogies with the hope that learners will be motivated and automatically develop the intended knowledge and skills [4, 15]. Educators find themselves utilize instructional teaching methods (traditional) more often because it is cheap, easy to implement, can cover an extensive course content at once, and suitable for a large group of students [16]. Moreover, some tutors and or lecturers are not well prepared and guided by formal pedagogical guidelines to enhance learning processes among students. Most of them demonstrated teaching skills in the way they were taught when they were students than the way educational requirements recommend them to practice. Likewise, students are trained in such a way they associate teaching and learning as the process, which involves preparing for a test or earning a grade [8].

However, scholars have noted that competency-based curricula aligned with facilitation in the problem-based environment, has been seen to be a robust educational solution [1, 4, 17]. PBL is a teaching pedagogy that uses real-world problems as the motivator of student’s self-directed learning [18]. The approach has an emphasis on academic motivation and knowledge construction rather than knowledge transmission [19]. Vygotsky [20] uncovered that students can build up new knowledge from their existing one (zonal of proximal development) while scaffolding being the support strategy to help them achieve their significant developmental potentials. Students exposed to the PBL are given opportunities to explore, investigate, analyze, synthesize, and carry out experiments and eventually reach their conclusions while the instructor’s roles are just to facilitate, direct, guide, and assist students to learn [21].

All the same, the PBL pedagogy requires instructors to play a role of posing questions to the entire class, and assign students to work in teams, and reach an agreement on their answers, which then they share in the entire class [1, 22, 23]. However, what is lacking is to understand how can problem-based facilitatory pedagogy be designed, implemented, and work in low-resource countries such as Tanzania in improving academic motivation among nursing students [17]. There are scarce scholarly works about the subject in Tanzania because little has been done to demonstrate the means through which Tanzanian nursing training institutions are actively implementing PBL pedagogy [14]. The current study aimed at determining the effect of problem-based facilitatory pedagogy on motivation to learn among undergraduate nursing students in higher Tanzanian training institutions. The study was guided by specific objectives including determining the effect of the problem-based facilitatory pedagogy on the level of intrinsic motivation, extrinsic motivation, and Amotivation to learn among undergraduate nursing students in Tanzanian higher learning institutions.

The null hypothesis was used to determine the effect of the independent variable over the outcome variable, which stated: “there was no significant difference in the levels of motivation to learn between nursing students exposed to the problem based facilitatory pedagogy and their counterparts in the control group (lecture-based learning method) in Tanzanian higher learning institutions.”

## Methods

### Study design and approach

A pre-post controlled quasi-experimental study design with a quantitative research approach. The study included consented undergraduate nursing students with steady class attendances. Recruitment of the study participants was done in early February 2018 before baseline data collection. A cluster-randomized educational institution via coin tossing (five hundred Tanzanian coins) was determined by the research assistants to allocate the sampled institutions into either be in an intervention or a control group. The Head of the coin was assigned to an intervention and the second side of the coin (with Buffalo image) to the control group. The researcher and research assistants did the tossing of the coin and identified the sides respectively.

Participants’ social demographic and academic characteristics were used to match them to ensure their similarities at baseline. As it has been used by previous studies of the same methodological approaches [1, 4], WinPepi Software program version 11.65 was considered reliable to be used in this study. The ratio of sample size (*n* = 401) was B:A = 1:2 with 134 participants in the intervention and 267 participants in the control group of which a proportionate formula $\left( ni= Pi\times \frac{Pi}{n}\right)$ was used to determine participants’ distribution at classroom levels. As shown in Table 1, participants in the intervention and those in the control group did the same pre-test and post-test respectively. However, they were treated differently whereas participants in an intervention group learned the conflict resolution content using a problem-based facilitatory pedagogy while those exposed in the control group learned the same content using a lecture-based learning (LBL) method.

**Table 1:** Summary of a Study Design

***Activity***	***Intervention group***	***Control group***
Pre-test (Baseline)	**√**	**√**
Intervention (Mid-line)	*Problem-based Facilitatory Pedagogy*	*Lecture-based Learning Method*
Post-test (End-line)	**√**	**√**

***Source:*** *Researcher’s Plan (2018)*

### Data collection process

The researcher and research assistants identified the study participants in the sampled study centers and put them in a separate and quiet room to ensure privacy and confidentiality during the filling process of questionnaires. Brief instructions on how to fill the questionnaires were provided among the study participants before distributing copies of the questionnaires. The researcher and assistants supervised the process and responded to participants’ queries throughout the process before collecting questionnaires. All participants answered the same questions before and after an intervention.

### Data collection tools

The instrument used for data collection was a structured Questionnaire titled Academic Motivation Scale (AMS-HS 28). A tool was adopted from AMS – College Version 1993 [24] having a Cronbach’s Alpha of 0.84. It has once been validated by Haugan et al., [25] and used by Millanzi et al.*,* [4] in Tanzania. The tool was used for assessing levels of intrinsic motivation (towards knowledge, accomplishments, and stimulation), extrinsic (introjected and identified regulation), and amotivation among undergraduate nursing students. The AMS-HS had 28 items with 140 scores on a 5-point point Likert scale. Scale 1 = does not correspond at all, 2 = correspond a little, 3 = corresponds moderately, 4 = corresponds a lot and 5 = corresponds exactly. Part A of the instrument elicited information about demographic data of the study participants including their age, sex, education level, accommodation status, and marital status. Other important participants’ characteristics included their interest status to join nursing, reasons to join it, their satisfaction status to nursing course, learning benefits, and learning difficulties they encountered during their learning processes. Part B elicited information about levels of motivation to learn adopted by students in their learning processes. This part covered three aspects (Intrinsic motivation, extrinsic motivation, and Amotivation to learn).

Intrinsic motivation was assessed by using a twelve [12] 5-point Likert (≥6 scores were defined as intrinsically motivated) scale items, extrinsic motivation twelve [12] items (≥6 scores were defined as extrinsically motivated), and Amotivation to learn 4 items (≥2 scores were defined as intrinsically motivated). The overall motivation to learn among the study participants was then computed that had a cut-off point set at 70 scores from 140 total scores of the scale items. The study participant, who scored ≥70 of the scale items, was defined as motivated to learn otherwise not.

#### Development and prototyping of the research conflict resolution material in the nature of problem-based Facilitatory pedagogy design

**Fig. 1:**
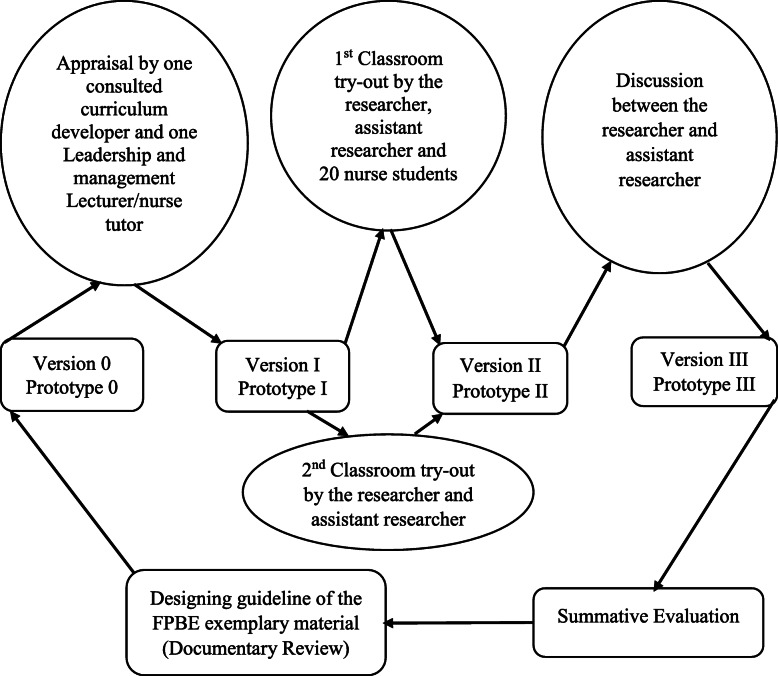
A flow chart showing Development, Prototyping style and Classroom Tryouts of the Conflict resolution material in the nature of problem-based facilitatory pedagogy. Source: adopted and modified the idea of Mafumiko [1, 28]

The process of developing and prototyping the PBL materials adopted the conceptualized framework that was developed by Mafumiko [26] but also used by Millanzi et al.*,* [27]. As shown in Fig. 1, the development and prototyping procedures of conflict resolution material in the nature of problem-based facilitatory pedagogy followed four [4] phases including phase 0, I, II, and III. Phase 0 focused on reviewing various documents including online and off-line books, published scholarly nursing profession magazines, and other relevant materials. The reviewed materials made it possible for the development of draft “0” of the conflict resolution material (Prototype “0”). The developed draft was then shared with the experts including tutors/lecturers who had at least > 1-year working experience in teaching leadership and management content and 1 expert in curriculum development for their professional and technical inputs, advice, and comments on the material.

The revised version was exposed to phases “I”, where a first classroom try-out among 20 nursing students, a principal investigator, 1 research assistant participated (Prototype “I”). Comments from experts in the first classroom try-outs were addressed accordingly and resulted in the second version, which was subjected to the second (phase II) classroom try-out (Prototype “II”). The researcher addressed the comments from the second classroom try-out whereas together with the assistant researcher discussed and amended the inputs. The third version (phase III) was refined and subjected to the actual field-testing (Prototype “III”). Summative evaluation of the effectiveness of PBL material on the learning process and the academic motivation was determined through a student’s experiences inventory (SEI) developed by the researcher and the administration of posttest among nursing students.

#### The intervention (problem-based Facilitatory teaching sessions)

The intervention part adopted and modified the intervention flow pattern by Millanzi et al.*,* [1] who implemented research of the same nature among nursing students in Tanzania. The intervention implementation involved aspects such as an introduction and formulation of groups, problem presentation, solving, discussions and sharing, scaffolding and group facilitation, mode of assessments, and summary of the study activities between an intervention and the control group.

##### Introduction and formulation of learning groups

This was the first-day segment which was characterized by the introduction about the intervention process including descriptions of the objectives and the expected terminal behavior among students throughout in-out class learning activities. The researcher and assistants randomly assigned a maximum of 8 students in groups. Given a facilitator’s guidance, members of the formed groups had to appoint a chairperson and secretary among themselves and other members were supposed to actively take part in the assigned activities.

##### Problem presentation, solving, discussions, and sharing

Objectives of each session had to be reviewed by the researcher and assistants before their commencements. Classroom management and organization was assured through getting members in their group seat in the round to promote eye contact and enhance the easy flow of discussions through sessions. The developed paper-based conflicts-based scenario among nurses at working places was shared with each group. Debriefing of how to address them through learning activities was done by the researcher and assistants before allowing them to start addressing the assigned problems. Given a 30 min, students were instructed to identify the professional context of the problem, listing what they knew, what they did not know, what they needed to know and establish the issues to learn from the problem.

Nevertheless, facilitators’ roles were to guide students via clarifying, ranking, and assigning learning tasks to each member of the group that would enable them to find appropriate strategies to resolve problems. Within 60 to 120 min, nursing students were guided to identify and suggest with reasons, the available resources needed to resolve the assigned scenario. Moreover, a period of 1 week was given among students to explore possible conflict resolution strategies that would be channeled to the scenario, then be shared and discussed in the entire class during the next scheduled session. As a closure of each session, nursing students were required to communicate to the researcher and or assistants either by mobile texts, orally or in writings through email whenever they need any help or clarifications of what was learned in the past sessions or what was to be learned in the next session. Following sessions after the offered week served as a room for students to present, discuss, and share what did with their colleagues in the entire class.

##### Scaffolding and group facilitations

Facilitators of sessions were required to scaffold the learning areas that students could not manage by themselves and facilitate their learning activities by making the learning environment friendly to their learning activities i.e. Adequate supply of learning resources and material and enough space for them to discuss or move freely and without disturbances each other. Owing to the big class size, sometimes, facilitators of the sessions used group leaders to control and lead group members. However, a leader who was used had to be briefly instructed on how to act as facilitators during monitoring and controlling the learning activities among members in respective groups.

##### Mode of assessment

Both, formative and summative assessments were used to assess nursing students’ learning behaviors and session achievements. Peer assessment served as a formative assessment among students that helped to determine students’ interactions and abilities to evaluate one another. To evaluate the course PBL material implementation, the facilitator administered a student’s experience inventory (developed by a researcher) among randomly selected students after each session. Besides, the written posttest was used as a summative assessment to measure the end-line levels of academic motivation among nursing students.

### Summary of the study activities between an intervention and the control group

Table 2 presents a flow plan of intervention with the session’s implementation to the end-line data collection between the intervention and the control group (Table 2).

**Table 2:** Summary of the study activities between an intervention and the control group (*n* = 401)

***Intervention group***	***Control group***
134 study participants recruited	267 study participants recruited
Administered the AMS-HS questionnaires as a pre-test to establish baseline information	Administered the AMS-HS questionnaires as a pre-test to establish baseline information
Participants organized in groups of 5 to 8 members	Participants were not organized into groups
Each member of the formed group was assigned a role to play by himself or herself e.g. Chairperson, secretary, timekeeper, etc. and they exchanged the roles per each session activities	Participants were not assigned in groups and thus had no roles to play alternatively
Participants were exposed to two sessions lasting for 90 min each per week according to the institutional schedule. 5 min were reserved for a break and 10 min for a facilitator to summarize the learned topic and respond to students’ queries per each session	Participants were exposed to two sessions lasting for 90 min each per week according to the institutional schedule. 5 min were reserved for a break per each session. No summary of the learned topic and respond to students’ queries from the facilitator per each session. “THANK YOU FOR YOUR ATTENTION” was the end sentence from the facilitator per each session
Exposed to nursing-based conflict resolution content through problem-based facilitatory pedagogy	Exposed to nursing-based conflict resolution content through lecture-based learning pedagogy
Participants in their respective groups were exposed to conflict scenario to study and find appropriate strategies to resolve it	Participants learned nursing-base conflicts at working place through facilitator-led PowerPoint presentations, question and answers, buzzing
A representative from each group had to share and defend their work in front of the entire class	Participants had opportunities to ask and answer questions from the facilitator
Participants in their respective groups were required to present and defend a homework activity of the previous session in the entire classroom before the commencement of another day’s session	Participants were required to answer questions from the facilitator about the previous session before the commencement of another day’s session
Participants were required to note-keep (establish records) of what is learned and performed per each session so that they could develop a summary of session activities by the end of all sessions	Participants had no opportunity. They just write summaries of what they learned for their read.
Two to three randomly selected participants had to share their experiences of the session activities, time, content dosage, and their opportunities to learn	Participants had no such opportunity. They had to disport once the session is over.
Administered the AMS-HS questionnaires as post-test to establish baseline information	Administered the AMS-HS questionnaires as post-test to establish baseline information

***Source:*** *Researcher’s plan (2018)*

### Data analysis

The study performed statistical analyses using the Statistical Product for Service Solutions (SPSS) version 23. Descriptive statistics through chi-square and cross-tabulation established participants’ socio-demographic profiles and determined the relationship between categorical variables. Binary and multinomial logistic regression analyses were performed to determine the association between variables. The confidence interval (CI) was set at 95% whereas the probability value of ≤0.05 was considered to be statistically significant. The power of the study was set at 80% of demonstrating the effectiveness of an intervention.

## Results

### Demographic characteristics of the study participants

Findings in Table 3 show that 65.8% of the study participants were males. Almost 73.6% of the study participants had ages ranging between 25 and 29 years. Moreover, 69.3% of the study participants lived in-campus. No statistically significant difference was observed in their gender, age, and marital status distributions between groups (*p* > 0.05). However, a significant difference was observed in the accommodation status of the participants between the two groups (*p* < 0.01) (Table 3).

**Table 3:** Demographic characteristics of the study participants between the Intervention and Control group (*n* = 401)

***Variable***	***Intervention***	***Control***	***P-value***
***Sex***	**n(%)**	**n(%)**	
Males	83 (61.9%)	181 (67.8%)	0.244
Females	51 (38.1%)	86 (32.2%)	
***Age***			
< 24 yrs.	6 (4.5%)	25 (9.4%)	
25–29 yrs.	100 (74.6%)	195 (73.0%)	0.192
> 30 yrs.	28 (20.9%)	47 (17.6%)	
***Marital status***			
Single	123 **(**91.8%)	248 **(**92.9%)	
Married	11 (8.2%)	19 (7.1%)	0.695
***In campus***			
Yes	43 **(**32.1%)	235 **(**88.0%)	
No	91 (67.9%)	32 (12.0%)	0.001

***Source:*** *Field Data (2019)*

### Other important participants’ characteristics (interest, reasons, satisfaction, learning benefits, and learning difficulties), which could influence motivation to learn

As shown in Tables 4, 73.8% of the study participants were interested to join the nursing profession and its programs while 52.4% of a sample chose nursing programs, as their first “own choice”. However, almost 20.9% of the study participants chose due to parent’s/peer pressure. Moreover, findings revealed that 75.3% of the participants were satisfied with nursing courses being taught to them whereas, 84.0% of them agreed that the teaching and learning practices were of a benefit to their academic achievements and career path. Nevertheless, 30.7% of the study participants reported that they experienced some difficulties in comprehending course contents due to their complexity, while 25.9% of a sample reported experiencing limited time to comprehend the course contents taught and participants who experienced difficulties in accessing learning materials constituted of 20.0%. Other findings were found as shown in the table (Table 4).

**Table 4:** Distributions of other important participants’ characteristics which were believed to influence academic motivation between the Intervention and Control group (*n* = 401)

***Variable***	***Intervention***	***Control***	***χ***^***2***^ ***P-value***
	***n(%)***	***n(%)***	
***Interest***			
Yes	92 (68.7%)	204 (76.4%)	*χ*^*2*^ *=* 2.771^a^
No	42 (31.3%)	63 (23.6%)	0.096
***Reasons to choose nurse***			
Own choice	71 **(**53.0%)	139 **(**52.1%)	
Parent’s/peer pressure	29 (21.6%)	55 (20.6%)	*χ*^*2*^ *=* 0.430^a^
Easier to get a job	24 (17.9%)	48 (18.0%)	0.934
Entry qualifications	10 (7.5%)	25 (9.4%)	
***Satisfaction***			
Yes	78 **(**58.2%)	224 **(**83.9%)	*χ*^*2*^ *=* 31.60^a^
No	56 (41.8%)	43 (16.1%)	0.001
***Learning benefits***			
Agreed	104 **(**77.6%)	233 **(**87.3%)	*χ*^*2*^ *=* 6.200^a^
Disagreed	30 (22.4%)	34 (12.7%)	0.013
***Learning difficulties***			
Difficult accessing updated learning materials	24 **(**17.9%)	56 **(**21.0%)	
Complex course contents	49 (36.6%)	74 (27.7%)	*χ*^*2*^ *=* 9.665^a^
Inadequate support from lecturers	18 (13.4%)	37 (13.9%)	0.046
Limited time	25 (18.7%)	79 (29.6%)	
No conducive environment	18 (13.4%)	21 (7.9%)	

***Source:*** *Field Data (2019)*

### Overall levels of motivation to learn and its subscales among nursing students

Findings of the levels of motivation to learn among nursing students were presented based on the overall motivation and its domains including intrinsic motivation (knowing what to learn, an accomplishment of learning tasks, and experiencing stimulation to learn), extrinsic motivation (identification of what to learn, introjection to learn, and regulation of motivation to learn), and amotivation to learn. The posttest findings in Table 5 indicate that 70.3% (*n* = 282) of the study participants demonstrated motivation to learn contrary to 34.9% (*n* = 140) at baseline. Additionally, there was a statistically significant gain of posttest motivation to learn among nursing students between motivation subscales of which 74.3% (*n* = 289) of participants demonstrated intrinsic motivation to learn. On the other hand, findings demonstrated that 64.3% (*n* = 258) of the study participants demonstrated extrinsic motivation to learn whereas 36.0% (*n* = 144) participants demonstrated amotivation to learn when assessed at post-intervention (Table 5).

**Table 5:** Overall Levels of Motivation to Learn and its Subscales among Nursing Students (*n* = 401)

***Variable***	***Pre-test***	***Posttest***
	***n(%)***	***n(%)***
Overall Motivation to Learn	140 (34.9%)	282 (70.3%)
Intrinsic Motivation to Learn	103 (25.7%)	289 (74.3%)
Extrinsic Motivation to Learn	143 (35.7%)	258 (64.3%)
Amotivation to Learn	257 (64.0%)	144 (36.0%)

***Source:*** *Field Data (2019)*

### Factors related to the effect of an intervention on the overall motivation to learn, among undergraduate nursing students between groups

Chi-square test and cross-tabulation findings in Table 6 point out that out of 282 (70.3%) of the study participants who were motivated to learn, 95.5% (*n* = 128) were those exposed in the intervention against 57.0% (*n* = 154) participants in the control group. It was found that an intervention (Problem-based facilitatory) pedagogy (*χ*^*2*^ = 7.041^a^, *p* < 0.01), accommodation (*χ*^*2*^ = 11.421^a,^ *p* < 0.01), and the reasons which made students join nursing programs (*χ*^*2*^ = 9.903^a^, *p* < 0.05) were significantly related to the overall motivation to learn among nursing students respectively. Other variables did not show a statistically significant relationship with the outcome variable as shown in the table (Table 6).

**Table 6:** Factors related to and the effect of Intervention on the overall Motivation to Learn among undergraduate nursing students between groups (*n* = 401)

***Variables***	***Motivation to Learn***		*χ*^*2*^ ***P-value***
	***Yes***	***No***	
	***n(%)***	***n(%)***	
***Groups***			
Intervention	128 (95.5%)	6 (0.5%)	*χ*^*2*^ = 7.041^a^
Control	154 (57.0%)	116 (43.0%)	0.008
*Gender*			
Males	202 (67.6%)	62 (60.8%)	*χ*^*2*^ = 1.552^a^
Females	97 (32.4%)	40 (39.2%)	0.213
***Age***			
< 24 Yrs.	20 (6.7%)	11 (10.8%)	
25–30 Yrs.	220 (73.6%)	75 (73.5%)	*χ*^*2*^ = 2.316^a^
> 30 Yrs.	59 (19.7%)	16 (15.7%)	0.314
***Marital status***			
Singles	278 (93.0%)	93 (91.2%)	*χ*^*2*^ = 0.356^a^
Married	21 (7.0%)	9 (8.8%)	0.551
***Accommodation status***			
In campus	209 (69.9%)	69 (67.6%)	*χ*^*2*^ = 11.421^a^
Off-campus	90 (30.1%)	33 (32.4%)	0.023
***Interest***			
Yes	223 (74.6%)	73 (71.6%)	*χ*^*2*^ = 0.357^a^
No	76 (25.4%)	29 (28.4%)	0.550
***Satisfaction***			
Yes	227 (75.9%)	75 (73.5%)	*χ*^*2*^ = 0.234^a^
No	72 (24.1%)	27 (26.5%)	0.629
***Reasons for choosing to nurse as a career***			
Own choice	162 (54.2%)	48 (47.1%)	
Parents/peer pressure	52 (17.4%)	32 (31.4%)	*χ*^*2*^ = 9.903^a^
Easier to get a job	59 (19.7%)	13 (12.7%)	0.019
Entry qualifications	26 (8.7%)	9 (8.8%)	
***Learning difficulties***			
Inadequate and difficulty in accessing updated learning materials	61 (20.4%)	19 (18.6%)	
Complex course contents	92 (30.8%)	31 (30.4%)	*χ*^*2*^ = 1.209^a^
Inadequate support from lecturers	43 (14.4%)	12 (11.8%)	0.877
Limited time	76 (25.4%)	28 (27.5%)	
No conducive environment	27 (9.0%)	12 (11.8%)	

***Source:*** *Field Data (2019)*

### The effect of an intervention on the overall motivation to learn among undergraduate nursing students between groups

Binary and multinomial logistic regression was done to determine the extent to which the intervention and the reasons that made nursing students join nursing programs had in motivating them to learn. Findings in Table 7 indicate that participants in an intervention group were 1.720 (AOR) times more likely to be motivated to learn against participants in the control counterparts (*p* < 0.05, CI: 1.122, 2.635). On the other hand, participants who were living on the campus were 0.591 (AOR) times less likely to be extrinsically motivated than the participants who were living out campus (*p* > 0.05, CI: 0.349, 1.002). However, the reasons that made nursing students join nursing programs did not influence motivating students to learn when adjusted to other variables (*p* > 0.05) (Table 7).

**Table 7:** Binary, and multinomial logistic regression to determine the effect of an Intervention on Motivation to Learn among undergraduate nursing students, between groups (*n* = 401)

***Variables***	***OR***	***95% CI***		***P-value***	***AOR***	***95% CI***		***P-value***
		***Low***	***Upper***			***Low***	***Upper***	
***Groups***								
Intervention	1.729	1.130	2.646	0.012	1.720	1.122	2.635	0.013
Control **(Ref)**								
***Accommodation***								
In campus	0.472	0.3.4	0.732	0.001	0.591	0.349	1.002	0.051
Off-campus **(Ref)**								
***Reasons for choosing to nurse as a career***								
Own choice	1.168	0.513	2.662	0.711	1.214	0.528	2.787	0.648
Parents/peer pressure	0.562	0.234	.1.352	0.198	0.578	0.239	1.402	0.225
Easier to get a job	1.571	0.597	4.132	0.360	1.635	0.616	4.337	0.324
Entry qualification **(Ref)**	0^b^	.	.			.	.	.

***Source:*** *Field Data (2019)*

## Discussion

### Socio-demographic characteristics of the study participants

The current study noticed many male students (65.8%) joined the nursing program as their first choice compared to females. It is interesting to note that the observed finding was one of the successes the nursing profession is earning in its transition from the past, present, and future. The findings imply that males are currently motivated to join the nursing program as compared to the past where female nursing students were many. However, these findings do not line up with those found by Kusumawaty, Kumara, Emilia & Haryanti [29] and Sabzevari, Abbaszade & Borhani [30], that showed a majority of females selected to join nursing as their priority than males. The mismatch of findings between the two studies would probably due to many of the previous studies were done a long time ago than the current study, which was conducted within the expansion of the nursing profession position and market in the globe.

Nevertheless, the day-to-day awareness creation interventions and programs about the nursing profession and its career paths through various media could contribute to the variation of findings between the two studies above. Its advanced roles among nurses, the breakthrough of a persisted myth that nursing was for females and the need for more educated and qualified nursing professionals, and its potential contribution to the individual and community well-being have currently attracted more people to join it including males. It is worth to observe both females and males select nursing as their pathway to advance their educational and professional careers, for the sake of serving the community. On the other hand, it was worth it too to observe a great number of men joining the nursing profession as this would clear out the belief, which has existed for a long time that nursing was for females.

### Overall motivation to learn and its subscales among undergraduate nursing students

Based on the findings presented above, it has been observed that with the control of other factors, problem-based facilitatory pedagogy was a predictive factor in enhancing motivation to learn among nursing students. Nursing students would demonstrate the ability to know what to learn, accomplish the learning activities, and experience stimulation to learn more and more. Students exposed to the intervention-demonstrated pleasure to learn as they felt that teamwork and being occupied with learning tasks during, after sessions were very important, and could contribute something to their academic progress.

On the other hand, extrinsic motivation is an aspect that defines the ways students are motivated to learn through the influence of external stimuli such as environments, peer pressures, punishments, and rewards were also studied. Nursing students who were exposed to the problem-based facilitatory pedagogy were less times likely to be extrinsically motivated to their learning process as compared to their counterparts under the lecture-based learning pedagogies who demonstrated motivation to learn in the presence of external stimuli commonly for being recognized, rewarded, and to be identified by their colleagues that they know.

Contrary to the effect of an intervention on motivation to learn among nursing students, is well accommodated by the training institutions, and individual student’s reasons to join nursing programs were found to be protective factors for them to be motivated to learn. The status of extrinsic motivation was also observed among nursing students who were living n campus. They were noted to be less times likely to develop extrinsic motivation to learn against students who were living off-campus. The majority of nursing students in the control group demonstrated to be motivated to learn just for being identified or recognized by others that they were able to know and solve issues. They demonstrated abilities to adopt values or attitudes they were impressed with for other famous or intelligent people for them to be accepted by others in their learning process. Moreover, nursing students in the control group were highly motivated to learn owing to the institutional regulations, principles, order, or rules.

Amotivation to learn among nursing students was measured to determine what discouraged them to learn. Findings demonstrated that participants who were exposed to problem-based facilitatory pedagogy were less likely demotivated in their learning processes as compared to their counterparts in the lecture-based learning pedagogies. The finding implied that the effect of the intervention and its associated setups and operationalization was significant than the lecture-based learning approach. Motivation has always been the central issue in nursing education and even being referred to as the most complex and challenging issue, facing educators, and students today. Various teaching modalities are tested in different settings and programs to help motivate students to learn with minimal support from educators.

Tallying with the findings of this study, Gaber et al.*,* [10] found that if well structured, problem-based learning could enhance learning force within a student. An internal force to learn within an individual was observed in their study to be potential in influencing and directing behavior and willingness among students to put efforts into achieving a goal or reward through decreasing their tension caused by their social and academic needs. Based on these facts, the researcher observed that problem-based teaching and learning pedagogy could positively make nursing students intrinsically motivated to learn and thus, become autonomous learners.

Moreover, the findings of this study are not new as they link with those found by Khamoushi et al.*,* [8], which revealed low academic achievements of undergraduate nursing students, to be attributed to the type of teaching pedagogies that educators used. The more the didactic teaching methods were used, the more students were demotivated to their learning process when compared to the usage of constructive teaching pedagogies. They then concluded that there was a necessity to respond to the gap appropriately to improve student’s academic achievements.

#### Limitation of the study

During the implementation phase of this study, group leaders were trained to act as facilitators. This would affect their full participation during in-out classroom activities, solving the presented problems in particular. Using group leaders would even make their colleagues not to take into serious their learning roles. The use of a quasi-experimental study design would influence selection bias among the study participants as it lacks randomization procedures. Lack of randomization procedures would make it difficult representation and generalizability the study findings.

## Conclusions

This study builds on and extends the earlier research findings on the effects of problem-based facilitatory teaching and learning processes. The findings of this study demonstrate that a pedagogical innovation in the nature of collaborative learning from a problem-based facilitatory pedagogy among nursing students promises to improve their motivation to learn than the predominantly use of lecture-based learning approaches. The effect of problem-based facilitatory teaching and learning pedagogy provided opportunities for self-directed and peer group learning interactions among nursing students. The opportunities empowered students to the level that they were able to view and share learning pathways among themselves without fear or feeling shy. This was done through the vantage point of problem identification that enabled them to propose learning issues, practice knowledge research and sharing, and then revisit the scenario to solve problems.

The findings indicate that problem-based teaching and learning pedagogy can positively influence the levels of intrinsic motivation to learn among students. The elements of the problem-based facilitatory pedagogy, which were used in the current study, have the potential contributions to the development of competent graduate nurses. The study concludes that the implementation of problem-based facilitatory pedagogy is feasible and practical in Tanzanian perspectives. It appears to be worth considering integrating and implementing it in the nursing curriculum to enhance a formal shift from the use of traditional teaching and learning approaches and try modern participatory ones to make nursing students intrinsically motivated to learn nursing programs.

## Data Availability

Data are available on request at walter.millanzi@udom.ac.tz or wcleo87@gmail.com because further analysis of other variables are being processed.

## Abbreviations

AM: Amotivation
AI: Auditing Inventory
AMS: Academic Motivation Scale
AOR: Adjusted Odds Ratio
CI: Confidence interval
EM: Extrinsic Motivation
EMI: Extrinsic Motivation Identified
EMIT: Extrinsic Motivation Introjected
ETR: Extrinsic Motivation external Regulation
FPBE: Facilitation in a Problem Based Environment
IM: Intrinsic Motivation
IMC: Intrinsic Motivation towards Accomplishment
IMK: Intrinsic Motivation to Know
IMS: Intrinsic Motivation to experience Stimulation
IRRC: Institutional Research Review Committee
PBL: Problem-based learning
PhD: Doctor of Philosophy
QMLSN: Questionnaires on Motivation to Learn Strategies in Nursing
SD: Standard Deviation
SEI: Student’s Experience Inventory
SPSS: Statistical Product for Service Solutions
UDOM: The University of Dodoma
WHO: World Health Organization
